# Contribution of fine particulate matter to present and future premature mortality over Europe: A non-linear response

**DOI:** 10.1016/j.envint.2021.106517

**Published:** 2021-08

**Authors:** Patricia Tarín-Carrasco, Ulas Im, Camilla Geels, Laura Palacios-Peña, Pedro Jiménez-Guerrero

**Affiliations:** a Department of Physics, Regional Campus of International Excellence Campus Mare Nostrum, University of Murcia, 30100 Murcia, Spain; b Aarhus University, Department of Environmental Science, Frederiksborgvej 399, DK-4000 Roskilde, Denmark; c Biomedical Research Institute of Murcia (IMIB-Arrixaca), 30120 Murcia, Spain

**Keywords:** Air pollution, Human health, Fine particulate matter, Climate penalty, Premature deaths, Cardiovascular diseases

## Abstract

The World Health Organization estimates that around 7 million people die every year from exposure to fine particles (PM_2.5_) in polluted air. Here, the number of premature deaths in Europe from different diseases associated to the ambient exposure to PM_2.5_ have here been studied both for present (1991–2010) and future periods (2031–2050, RCP8.5 scenario). This contribution combines different state-of-the-art approaches (use of high-resolution climate/chemistry simulations over Europe for providing air quality data; use of different baseline mortality data for specific European regions; inclusion of future population projections and dynamical changes for 2050 obtained from the United Nations (UN) Population Projections or use of non-linear exposure–response functions) to estimate the premature mortality due to PM_2.5_. The mortality endpoints included in this study are Lung Cancer (LC), Chronic Obstructive Pulmonary Disease (COPD), Cerebrovascular Disease (CEV), Ischemic Heart Disease (IHD), Lower Respiratory Infection (LRI) and other Non-Communicable Diseases (other NCDs). Different risk ratio and baseline mortalities for each disease end each age range have been estimated individually. The results indicate that the annual excess mortality rate from fine particulate matter in Europe is 904,000 [95% confidence interval (95% CI) 733,100–1,067,800], increasing by 73% in 2050s (1,560,000; 95% CI 1,260,000–1,840,000); meanwhile population decreases from 808 to 806 million according to the UN estimations. The results show that IHD is the main cause of premature mortality in Europe associated to PM_2.5_ (around 48%) both for the present and future periods. Despite several marked regional differences, premature deaths associated to all the endpoints included in this study will increase in the future period due to the climate penalty but especially because of changes in the population projected and its aging.

## Highlights

• Premature mortality associated to fine particles (PM_2.5_) over Europe is estimated.
• Excess mortality rate from PM_2.5_ in Europe is 904,000 premature deaths/year.
• This mortality rate will increase by 73% in the year 2050 under RCP8.5 scenario.
• Increase in premature mortality is associated fundamentally to the aging of population.

## 1 Introduction

As stated by the World Health Organization (WHO), 9 out of 10 people breathe polluted air (WHO, 2018a, WHO, 2018, Chen, 2019, Kadaverugu et al., 2019). This means that most of the world population is threatened by this “invisible killer” (O’Connor, 2019). The effects of air pollution on human health are plentiful, leading to an increase of the morbidity, a general decrease in the life expectancy, or even causing acute deaths (Brook et al., 2010, Héroux et al., 2015) or premature deaths attributable to non-communicable diseases (NCDs) (Nghavi et al., 2017). NCDs (also called chronic diseases) are long-duration diseases, resulting from a combination of some factors (genetic, physiological, environmental and behavioural) and kill 41 million people each year (71% of global deaths) (WHO, 2018b). 48% of deaths caused by NCDs are considered premature because they occur before the age of 70 (Magnusson, 2019).

Different air pollutants have diverse effects on health, but the pollutant with the most important effect is fine particulate matter (particulate matter with a diameter under 2.5 µm, PM_2.5_) (Burnett et al., 2018, Liang et al., 2018). The WHO estimates that around 7 million people die every year from exposure to fine particles (WHO, 2018a, WHO, 2018). In addition, there is enough evidence about the relationship between PM_2.5_ and diseases such as stroke, heart disease, lung cancer, chronic obstructive pulmonary diseases and respiratory infections, including pneumonia (e.g. Liu et al., 2009, Pope et al., 2009, Brook et al., 2010, Pope et al., 2011, Anenberg et al., 2014, Ford and Heald, 2016, Hvidtfeldt et al., 2019, Lelieveld et al., 2020; among many others). Not only short-term exposure has been associated with increases in daily mortality. Long-term exposure also leads to chronic effects on human health (e.g. Hoek et al., 2002, Krewski et al., 2009, Brook et al., 2010, Pope et al., 2011, Beelen et al., 2014, Bell et al., 2014, Burnett et al., 2014, Guan et al., 2019). In addition, these health damages and mortality cause important economic losses (e.g. Brandt et al., 2013a, Brandt et al., 2013b, Im et al., 2018). Recently, Tarín-Carrasco et al. (2019) estimated that premature deaths related to air pollution are the most important environmental problem in Europe with respect to costs (158 billion euro per year), increasing by 17% in the future RCP8.5 2071–2100 scenario only due to climate penalty.

When considering these multidisciplinary problems associated to fine particles, recent assessments indicate that the estimation of PM_2.5_ concentration is still intricate due to the need of high resolution data for establishing an accurate estimation of their levels on regional and local domains (e.g. Parvez and Wagstrom, 2020, Wolf et al., 2020). However, recent estimations of PM_2.5_ and their health effects still rely on global data at very coarse resolution (over 1°). For instance, Silva et al. (2016) used global model data varying ranging from approximately 2° to 5° (regridding them to a common 0.5° horizontal grid) to estimate a global burden of 3.25 million deaths per year in 2100 associated to fine particulate matter. Lelieveld et al. (2020) used the EMAC model at T106 horizontal (grid spacing of about 1.1° latitude and longitude) for estimating a global excess mortality from all ambient air pollution of 8.8 million per year, being a factor of two higher than earlier estimates (e.g. Cohen et al., 2017 estimated the excess mortality burden as 4.2 million per year). In spite of this, Thompson et al. (2014) highlight a strong improvement in the estimations of the impacts of air pollution on human health when increasing the resolution up to 12 km (around 0.11°). Last, Chowdhury et al. (2020) use data with very high resolution (0.1°) from the Data Integration Model for Air Quality (DIMAQ) model (Shaddick et al., 2018) to estimate present ambient PM_2.5_ exposure at high resolution between 2010 and 2015. Henceforth, using high-resolution air quality data is essential for accurate estimates of the health impacts of PM_2.5_

In addition, the complexity of the problem increases for future climate change scenarios because of the uncertainties related to projections of temperature and precipitation, as well as wildfires and natural emissions (Jacob and Winner, 2009, Fuzzi et al., 2015, Fiore et al., 2015). Despite these uncertainties, an important number of works agree that climate change modifies the chemistry, transport and deposition of some pollutants, worsening regional air quality (e.g. Jacob and Winner, 2009, Jiménez-Guerrero et al., 2013a, Jiménez-Guerrero et al., 2013b, Liu et al., 2016) and in consequence, increasing premature deaths over Europe (e.g. Geels et al., 2015, Doherty et al., 2017, Im et al., 2018, Tarín-Carrasco et al., 2019) and over the entire world (e.g. Lelieveld et al., 2015, Silva et al., 2017, Kinney, 2018).

Hence, the objective of this study is to estimate the excess premature mortality associated to fine particulate matter over Europe due to different endpoints or causes of premature mortality. To cope with this objective: (1) the mortality rates for Lung Cancer (LC), Chronic Obstructive Pulmonary Disease (COPD), Low Respiratory Infections (LRI), Ischemic Heart Disease (IHD), cerebrovascular disease (CEV) and other Non-Communicable Diseases (other NDCs) associated to fine particles are estimated for a present period (1991–2010); and (2) the difference with the future (period 2031–2050) mortality rates caused by climate penalty (under the RCP8.5 scenario) and population dynamics is calculated considering changes in PM_2.5_ concentration and population. This contribution uses three state-of-the-art approaches combined to provide the estimation of the excess premature deaths associated to particulate matter: the use of high-resolution climate/chemistry simulations over Europe (0.11°) for providing air quality data; the use of different baseline mortality data for specific European regions; and the inclusion of future population projections and dynamical changes for 2050 obtained from the United Nations (UN) Population Projections.

## 2 Methodology

The excess premature mortality rates in Europe have been estimated by using exposure–response functions. Different methodologies, scenarios, periods and/or emissions are used in the scientific literature to estimate the impacts of outdoor pollution on human health. In the present contribution, two different non-linear functions were a priori considered for the calculation of premature mortality due to exposure to PM_2.5_, as indicated in the Supplementary Material (SM): the Integrated Exposure-Response (IER) model used by the Global Burden Disease (GBD); and the Global Exposure Mortality Model (GEMM) methodologies. With respect to IER, Burnett et al., 2014, Lelieveld et al., 2015, Cohen et al., 2017 or Liang et al. (2018), among others, used this methodology to calculate different cause-specific mortality all over the entire world or over regional areas such as Europe. The GEMM methodology was developed by Burnett et al. (2018) and was also used in Lelieveld et al. (2019). The description of both methodologies, together with a comparison of their results, is presented in the SM. The main differences between IER and GEMM are caused by the different individual cohort studies covered in each methodology. GEMM includes a higher number of cohorts exposed to a poor air quality with respect to those included in IER (Burnett et al., 2018). The updated literature included in GEMM and the higher number of cohort studies taken into account over areas with PM_2.5_ concentrations similar to the target area covered here (up to nearly 40 μg m^−3^ of PM_2.5_ as annual mean), conditioned the election of the GEMM methodology for estimating the premature mortality rates associated to PM_2.5_ over Europe.

### 2.1 Estimation of risk ratios and mortality due to exposure to PM2.5

As aforementioned, premature mortality is estimated using exposure–response functions describing the association between PM_2.5_ and non-accidental mortality. Eq. (1) denotes the exposure–response function followed in this work. This equation is based on epidemiological relationships between air pollution concentration and mortality; and is applied in each grid cell where information about air pollution and population is available (Lelieveld et al., 2015, Lelieveld et al., 2019):

(1) $$\Delta M\;=\;y_{0}\left[\left(\mathrm{RR}-1\right)\;/\;RR\right]\;Pop$$

where *ΔM* is premature mortality for each disease; RR is the risk ratio, which estimates the relative risk of exposure to a specific pollutant (see Equations SM.1 and SM.2 for further details); *y_o_* is the baseline mortality rate (GHDE, 2019); and *Pop* refers to the exposed population. *y_o_* varies for each mortality endpoint, age and European region and is estimated annually by the WHO for each gender. Sex mixing values used in the present study account for both male and female dwellers during the year 2010. RRs and hence excess premature mortality have been estimated for each endpoint and for different age groups: 25–29, 30–34, 35–39, 40–44, 45–49, 50–54, 55–59, 60–64, 65–69, 70–74, 75–79, +80 and all ages.

With respect to the studied pathologies, the GEMM methodology includes Lung Cancer (LC), Chronic Obstructive Pulmonary Disease (COPD), Cerebrovascular Disease (CEV), Ischemic Heart Disease (IHD), Lower Respiratory Infection (LRI). Also, the category of non-accidental diseases (defined as NCD + LRI) is included together with the so-called ‘other NCDs’, defined as the subtraction of the previous categories to NCD + LRI. Uncertainty ranges are expressed as the 95% confidence intervals (95% CIs). It should be emphasized that the 95% CI refers to statistical uncertainty associated with the epidemiological data, and not methodological uncertainty, including unaccounted confounding factors, assumptions about counterfactuals or limited representativeness of the hazard ratio functions (Lelieveld et al., 2020).

**Fig. 1:**
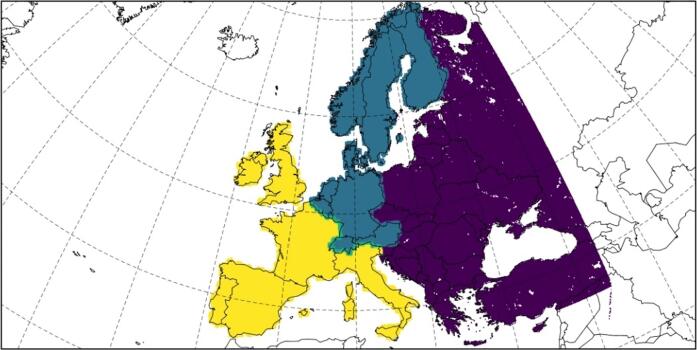
Target domain and European regions included in this contribution: Western Europe (yellow), Central Europe (blue) and Eastern Europe (purple). (For interpretation of the references to colour in this figure legend, the reader is referred to the web version of this article.)

In the present study, Europe was divided into three different regions (Fig. 1) after Couvidat et al., 2018, Pandolfi et al., 2018. Different values of the baseline mortality rate are used for each region, taking into account a number of different variables like the type of public health system, the economy of the country and the climatological characteristics, and is kept constant in the future scenario. The value of baseline mortality is similar for each country within each of the three specified regions.

Hence, the final regions (Fig. 1) are: (1) Western Europe: Portugal, Spain, France, Italy, United Kingdom and Ireland; (2) Central Europe: Denmark, Finland, Norway, Sweden, Austria, Belgium, Germany, Luxembourg, The Netherlands and Switzerland; and (3) Eastern Europe: Turkey, Belarus, Bulgaria, Czech Republic, Hungary, Poland, Republic of Moldova, Romania, Russian Federation, Slovakia, Ukraine, Estonia, Latvia, Lithuania, Albania, Bosnia and Herzegovina, Croatia, Greece, Montenegro, North Macedonia, Serbia and Slovenia.

### 2.2 Air pollution data

Air pollution data for estimating present and future mortality under a climate change scenario come from on-line climate/chemistry simulations. The WRF-Chem online-coupled climate/chemistry model (Grell et al. 2005) version 3.9.1.1 with a horizontal resolution of 0.11° (around 12 km) has been used under the umbrella of the REPAIR and ACEX projects (Palacios-Peña et al., 2020a, López-Romero et al., 2020) over the Euro-CORDEX (Jacob et al., 2020) domain. The physico-chemical configuration has been summarized in Table 1. The present reference period spans 1991–2010. This reference period has been selected as a historical reference period well established in the scientific literature for climate studies (e.g. Barry et al., 2018, Marsha et al., 2018, Beringer et al., 2020, among others). The future enhanced forcing scenario is represented by the period 2031–2050 under the RCP8.5 scenario developed by the Intergovernmental Panel on Climate Change (IPCC), which is at the top of radiative forcing scenarios among all the Representative Concentration Pathways (RCPs; Moss et al., 2010). The differences between these two runs will provide the changes in future air quality. Following the consolidated scientific literature, constant anthropogenic emissions for the present and future scenarios have been assumed in order to isolate the effects of climate change alone (e.g. Watson et al., 2016, Hong et al., 2019, Tarín-Carrasco et al., 2019).

**Table 1:** Physico-chemical parameterizations implemented in WRF-Chem simulations over Europe.

***Parameterization***	***Option***	***Reference***
**Physics**		
Microphysics	Lin	Lin et al. (1983)
Land Surface	NOAH	Tewari et al. (2004)
Longwave and shortwave radiation	RRTM	Iacono et al. (2008)
Cumulus	Grell 3D Ensemble	Grell and Dévényi (2002)
Planetary Boundary Layer	YSU	Hong et al. (2006)
**Chemistry**		
Aerosol	GOCART	Ginoux et al., 2001, Chin et al., 2002
Gas-phase	RACM-KPP	Stockwell et al., 2001, Geiger et al., 2003
Photolysis	Fast-J	Fast et al. (2006)
**Anthropogenic and Natural emissions**		
Anthropogenic emissions (constant)	ACCMIP	Lamarque et al. (2010)
Biogenic emissions	Model of Emissions of Gases and Aerosols from Nature model (MEGAN)	Guenther et al. (2006)
Dust emissions	GOCART	Goudie and Middleton, 2001, Goudie and Middleton, 2006 (further details in Palacios-Peña et al., 2019b)
Sea Salt emissions	GOCART	Chin et al. (2002)

Simulations were driven by the GCM CMIP5-experiment (Taylor et al., 2012). The r1i1p1 MPI-ESM-LR historical run (Giorgetta et al., 2012a) for the present period and the RCP8.5-forced r1i1p1 MPI-ESM-LR run (Giorgetta et al., 2012b) for the future scenario. The evolution of CO_2_, CH_4_, and N_2_O greenhouse gases in the regional model has been considered after Jerez et al. (2018). Vertical resolution is 29 vertical sigma levels with the top at 50 hPa. The simulated periods were split into 5-year periods continuously run with a spin-up period of 4 months, following the recommendations of Jerez et al. (2020). Due to the lack of dust concentrations in the boundary conditions, an outer domain with a spatial resolution of 1.32° covering the main source of Saharan dust (Goudie and Middleton, 2001, Goudie and Middleton, 2006) were used as in Palacios-Peña et al. (2019b). This outer domain was run with spectral nudging in order to maintain a more realistic synoptic situation.

The results of the modelling system presented here have been extensively evaluated in previous works (e.g. Palacios-Peña et al., 2019a, Palacios-Peña et al., 2020a,b). However, for the sake of clarity, a brief description of the evaluation results is included here. The aerosol particles in the simulations have been compared to both ground-level and remote-sensing aerosol optical depth (AOD) data from the Moderate Resolution Imaging Spectroradiometer (MODIS), finding underestimations in the simulations that can be related to the misrepresentation of black carbon coming from biomass burning and a misrepresentation of the aerosol vertical profile modelled in these simulations (Palacios-Peña et al., 2019a).

The evaluation results reveal negligible errors of the model over large areas of Europe but an underestimation over northern Germany and the central and western Mediterranean (around −0.2 to −0.4). Despite this underestimation, the spatiotemporal mean bias error and absolute error over the target domain are limited (−0.02 and 0.16), respectively, when evaluated against MODIS data (Palacios-Peña et al., 2020b).

### 2.3 Population data in the European domain

Population data for Europe have been taken from the gridded dataset of NASA SocioEconomic Data and Applications Center (SEDAC) (http://sedac.ciesin.columbia.eduhttp://sedac.ciesin.columbia.edu) *Basic Demographic Characteristics, v4.11* (SEDAC, 2019). These data provide the population density by age and gender for the year 2010 consistent with national censuses and population registers with a gridded resolution of 5 km × 5 km.

Population data were interpolated to the Euro-CORDEX working grid for consistency with the gridded air pollution data. With respect to the future population, a projection for the year 2050 has been estimated by using information from the Population Prospects from United Nations Organization (UN) Department of Economic and Social Affairs Population Dynamics (UN, 2019). This includes both an evolution of both the total national population and the age distribution.

**Fig. 2:**
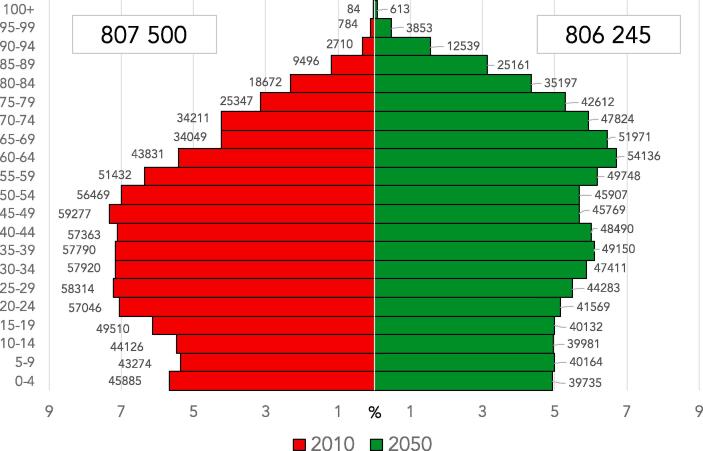
Population pyramid by age range (in thousands) for the year 2010 (red, left) and 2050 (green, right). x-axis represents the percentual contribution of each age range to the total population. (For interpretation of the references to colour in this figure legend, the reader is referred to the web version of this article.)

**Fig. 3:**
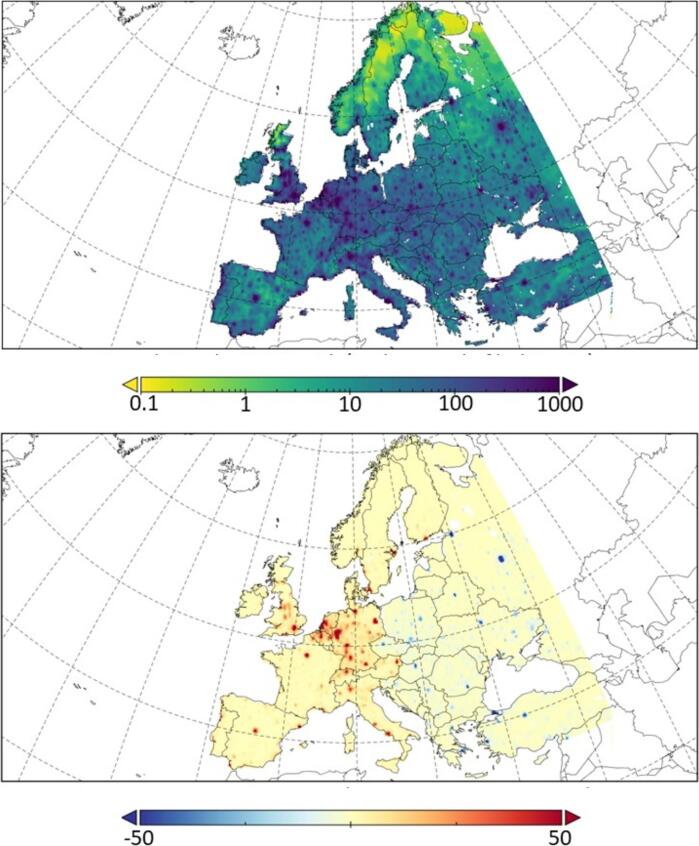
Population density (pop/km^2^) in each grid cell for the present scenario (top) and difference with UN-projected population in 2050 (bottom) over the European target domain (pop/km^2^).

The relative variation of the population from this dataset between 2010 and 2050 for each European country and age range was calculated in order to obtain the ratio of population for the future scenario (2050). The population pyramid both for the years 2010 and 2050 is presented in Fig. 2. This Figure indicates a slight decrease projected for the European population (808 vs. 806 million dwellers for present and future population, respectively), especially over Eastern Europe (Fig. 3). In addition, the projected data includes a higher population density over many urban areas, and a clear ageing of the European citizens. As an example, the population over 80 years (80+) barely represents 4% of the total European population nowadays, while it is expected to increase to over 9% in the projected UN 2050 estimations.

## 3 Results

This section presents the results obtained for excess mortality rates associated to fine particles by using the GEMM methodology (described in the SM). GEMM is applied to both present and future period). For the future scenario, the effects of the climate penalty and the evolution of the European population were considered.

### 3.1 Present and future concentration of fine particulate matter (PM_2.5_)

**Fig. 4:**
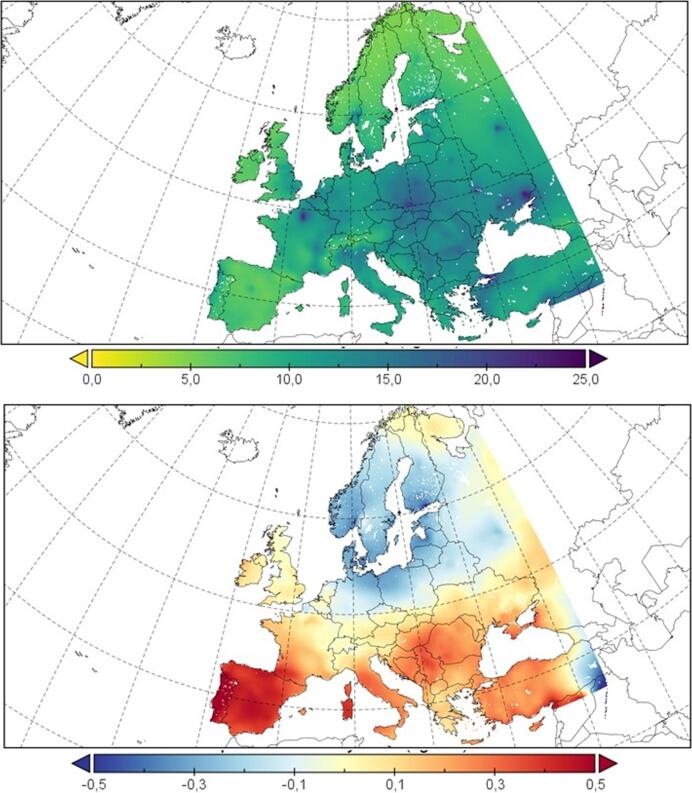
(Top) Annual mean PM_2.5_ concentration over Europe during the present climatic period (1991–2010). (Bottom) Difference in PM_2.5_ levels between the present and future period (2031–2050, RCP8.5) (mean for present minus mean for the future) (all units µg m^−3^).

Fig. 4 (top) shows the annual mean surface concentration of PM_2.5_ (averaged over the period 1991–2010) used for estimating the RRs and, therefore, *ΔM* in Eq. (1).

According to the Directive 2008/50/EC on ambient air quality and cleaner air for Europe, the PM_2.5_ limit value for the protection of human health is set to an annual mean concentration of 25 μg m^−3^. Eastern Europe is the area where the highest PM_2.5_ concentrations are estimated in the simulation for 1991–2010 (Fig. 4, top). In most areas, the level is below this limit value; however, in some hotspots like Paris (France), Krakow (Poland), Moscow (Russia) and the eastern part of Ukraine the concentration exceeds 25 μg m^−3^. Fig. 4 (bottom) shows the projected changes of air pollution associated to PM_2.5_ over Europe in 2031–2050 under the RCP8.5 scenario, where the strong south-north dipole pattern observed (increase of PM_2.5_ in southern Europe and decrease over northern areas) has been widely discussed elsewhere (e.g. Jiménez-Guerrero et al., 2013a, Jiménez-Guerrero et al., 2013b, Tarín-Carrasco et al., 2019). In particular, PM_2.5_ over the Iberian Peninsula is projected to increase by > 2 μg m^−3^ (annual mean) due to the climate penalty. Conversely, a decrease of the PM_2.5_ concentration (around 0.3 μg m^−3^) is simulated over northern Europe, that can be attributed to an increase of the precipitation over this area on the future projections (Jacob et al., 2018). This will entail a better air quality by enhancing the removal of particles (Hou et al., 2018). On the other hand, a reduction in precipitation is expected in southern Europe under the RCP8.5, hampering the wet scavenging and worsening air quality (Jiménez-Guerrero et al., 2013a).

### 3.2 Present (1991–2010) PM_2.5_-related premature mortality for Europe

**Fig. 5:**
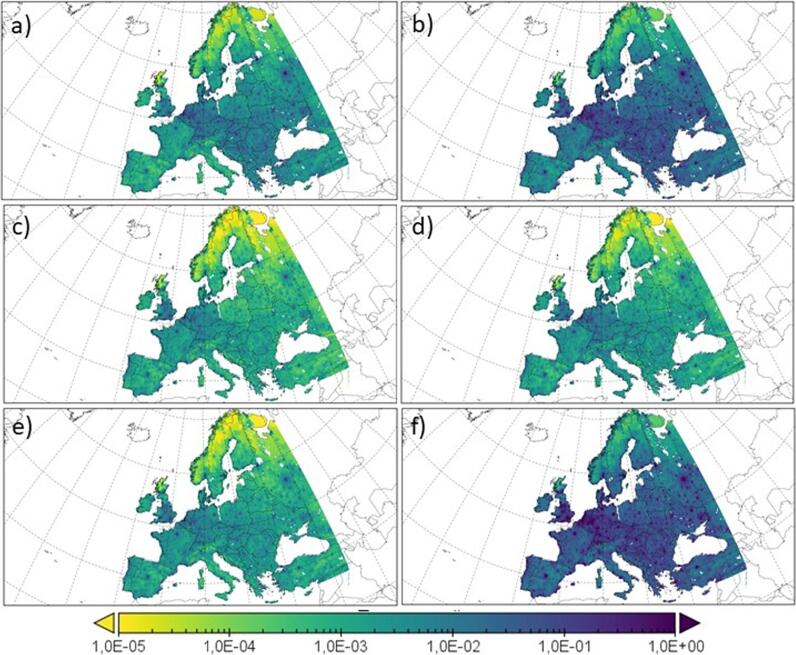
Estimation of annual premature deaths associated with PM_2.5_ exposure in Europe per km^2^. (a) CEV, (b) IHD, (c) COPD, (d) LC, (e) LRI and (f) All causes for the present period (1991–2010).

This section describes the spatial distribution of excess premature mortality associated to PM_2.5_ over Europe during the present period (1991–2010). The different endpoints studied in this contribution are shown in Fig. 5. Table 2 summarizes the results found regarding the different causes of premature deaths over the three different regions (Western, Central and Eastern Europe).

**Table 2:** Population and annual excess premature mortality for the present period (1991–2010) over Europe, shown by geographic region. Numbers in parenthesis indicate the 95% confidence interval (population in millions; endpoints and confidence interval in thousands).

	**Pop. (x10^6^)**	**CEV (x10^3^)**	**IHD (x10^3^)**	**COPD (x10^3^)**	**LC (x10^3^)**	**LRI (x10^3^)**	**Other NCD (x10^3^)**	**All (x10^3^)**
Western	248	8 (6.3–8.7)	41 (34.1–46.8)	9 (7.0–10.0)	13 (11.0–15.2)	11 (9.5–13.1)	84 (44.5–123.8)	166 (134.0–195.4)
Central	150	23 (19.1–26.2)	100 (83.7–114.8)	9 (7.1–9.9)	15 (12.7–17.5)	10 (8.7–12.1)	84 (44.2–123.0)	241 (194.7–283.7)
Eastern	409	59 (49.1–67.3)	294 (247.2–338.6)	11 (9.0–12.3)	18 (15.0–20.9)	20 (16.9–23.3)	95 (50.0–139.0)	497 (401.9–585.7)
**EUROPE**	**808**	**90 (75.5**–**103.5)**	**435 (365.4**–**500.3)**	**29 (24.3**–**33.4)**	**46 (38.5**–**52.9)**	**41 (34.3**–**47.2)**	**263 (144.6**–**386.6)**	**904 (733.1**–**1,067.8)**

The overall spatial distribution of the excess premature deaths is similar for all the different health endpoints. COPD (Fig. 5c), LC (Fig. 5d) and LRI (Fig. 5e) are the causes with a lower and less extended associated mortality over the domain (<1 case of premature death per km^2^). On a yearly basis, COPD totalizes 9,000 (95% CI 7,000–10,000) premature deaths per year in Western and Central Europe; and 11,000 (95% CI 9,000–12,300) over Eastern Europe. For LC, 13,000 (95% CI 11,000–15,200) annual premature deaths are estimated over Western Europe, 15,000 (95% CI 12,700–17,500) over Central Europe and 18,000 (95% CI 15,000–20,900) over Eastern Europe.

Last, LRI totalizes an excess of 11,000 (95% CI 9,500–13,100) premature deaths per year over Western Europe, 10,000 (95% CI 8,700–12,100) over Central Europe and 20,000 (95% CI 16,900–23,300) over Eastern Europe. Central Europe is the area where the highest number of cases per km^2^ was found (e.g. Benelux, the southern UK, Western Germany and some hotspot located in large urban areas). This highlights the combination of high PM_2.5_ levels and a high population density.

For the CEV distribution (Fig. 5a), 8,000 (95% CI 6,300–8,700) excess premature deaths per year are estimated in Western Europe, 23,000 (95% CI 19,100–26,200) over Central Europe and 59,000 (95% CI 49,100–67,300) over Eastern Europe (in addition to the aforementioned areas for Central Europe, some more zones in Eastern Europe, as Czech Republic, Slovakia or Hungary, have > 1 mortality case per km^2^).

Conversely, excess IHD premature mortality rate (Fig. 5b) totalizes 41,000 (95% CI 34,100–46,800), 100,000 (95% CI 83,700–114,800) and 294,000 (95% CI 247,200–338,600) per year in Western, Central and Eastern Europe). All causes of annual premature deaths (Fig. 5f) add up to 166,000 (95% CI 134,000–195,400), 241,000 (95% CI 194,700–283,700) and 497,000 (95% CI 401,900–585,700) in Western, Central and Eastern Europe, respectively. For IHD and All causes, > 6 premature deaths/km^2^ per year and > 10 premature deaths/km^2^ per year, respectively, were obtained. The spatial distribution of the mortality cases across Europe is quite similar as that reported by Lelieveld et al. (2019) using the same methodology. These authors estimated a total of 790,000 premature deaths per year over Europe; while Burnett et al. (2018) estimated 647,000 annual premature deaths associated to air pollution. These numbers are slightly lower than the total excess mortality rate of 904,000 (95% CI 733,100–1,067,800) presented in this study (+13% and +28% deaths per year in this work when compared to Lelieveld et al., 2019, Burnett et al., 2018; respectively). These differences can be ascribed to a number of factors, namely: the size of the domain, the population used in this work, the higher modelling resolution with respect to previous works (which makes the concentration in urban areas higher than that in the aforementioned contributions), different modelling setup, etc.

The distribution of all mortality endpoints follows the same pattern in Europe as in the previous studies, with the highest number of cases over Eastern and Central domains, in particular in the Benelux area. Also, several hotspots can be found in some European megacities as Madrid, Paris or Moscow. Northern Europe (Norway, Sweden and Finland) is the area with the lowest number of premature deaths for all the diseases covered in this work.

Summarizing, Eastern Europe is the area of most concern in terms of premature mortality associated to PM_2.5_ (Table 2 and Fig. 5). IHD is the pathology causing the highest number of premature deaths (294,000) per year in Eastern Europe. Over this area, COPD is the endpoint with the lower rate of excess premature deaths per year (11,000). A similar pattern is seen for Central Europe (the smallest region in terms of population), where IHD leads the number of premature deaths per year (100,000). Conversely, COPD is the pathology with the lowest contribution (9,000) to excess premature mortality rate. For Western Europe, the most noticeable difference is that the lowest rate of excess mortality cases per year are linked to CEV (8,000). Western Europe is the area with the lowest mortality rate (18% of the total European mortality, compared with 27% of Central Europe or 55% of Eastern Europe).

**Fig. 6:**
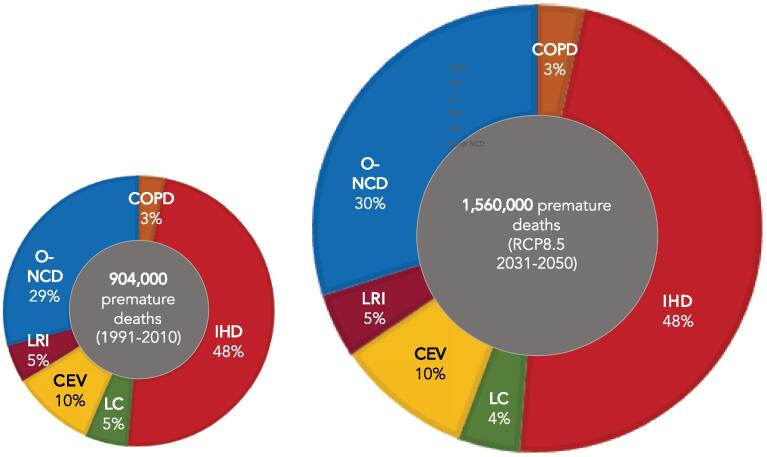
Percentages of premature mortality by disease in Europe for the present period (1991–2010, left) and future RCP8.5 scenario (2031–2050, right).

Fig. 6(left) displays the total ratio (%) of each mortality cause for the present-day conditions. 48% of the excess mortality rate is associated to IHD (435,000; 95% CI 365,400–500,300); being the pathology with the highest impact in the excess of total premature deaths. On the other hand, the cause with the lowest contribution to mortality rate from PM_2.5_ is COPD (29,000; 95% CI 24,300–33,400), which is about 3% of the total mortality associated to PM_2.5_ in Europe.

As commented before, these numbers are slightly higher than those by Lelieveld et al. (2019), who accounted for 64,000 CEV deaths per year from air pollution (90,000 in this contribution, +29%, 95% CI 75,500–103,500) and 313,000 related to IHD (435,000 here, +28). Hence, the main cause of mortality in Europe associated with the exposure to PM_2.5_ has to be sought in cardiovascular diseases (IHD + CEV), representing the 58% of the total premature deaths. Other NCDs are also important, since they represent the 29% of the total contribution to premature mortality (263,000 premature deaths per year, 95% CI 144,600–386,600).

Fig. 6(left) also reveals that LC and LRI represent around the 5% of mortality causes (46,000; 95% CI 38,500–52,900 for LC and 41,000; 95% CI 34,300–47,200 premature deaths per year), respectively. COPD totalizes just the 3% (29,000 deaths per year).

### 3.3 Future (2031–2050, RC8.5 scenario) PM_2.5_ premature mortality for Europe

The relationship between PM_2.5_ concentration in Europe and the different mortality causes under (1) a changing climate (RCP8.5 scenario); and (2) projected population was analysed. Overall, the number of excess premature deaths caused by PM_2.5_ will increase across all of Europe to 1,560,000 (95% CI 1,260,000–1,840,000) (+73% compared to the present estimation of 904,000). The results also show that the contribution of each endpoint to annual premature deaths associated to air pollution will keep barely altered in the future RCP8.5 scenario (Fig. 6, right), so the discussion conducted previously is also valid here.

**Fig. 7:**
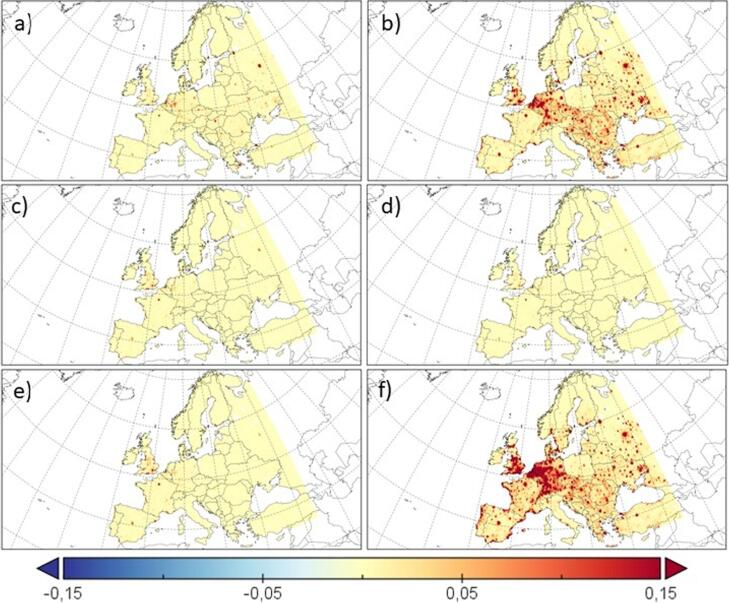
Differences between future (RCP8.5, 2031–2050) and present (1991–2010) annual premature mortality in Europe (premature deaths/km^2^) in both simulations. a) CEV, b) IHD, c) COPD, d) LC, e) LRI and f) All causes.

Fig. 7 indicates that the different causes leading to an excess in premature mortality associated to fine particulate matter are located in overpopulated areas with a high concentration of PM_2.5_. These areas match large European cities, Central Europe (mainly Benelux and Germany) and Eastern Europe.

The increase in the mortality rate associated to fine particles in the future (Table 3) can be ascribed to the increase of PM_2.5_ under the RCP8.5 scenario in southern countries together with the aging of the population (previously shown in Fig. 2).

**Table 3:** Estimation of annual premature mortality by disease for present (1991–2010) and future (RCP8.5, 2031–2050) periods (in thousands). Numbers in parenthesis indicate the 95% confidence interval.

	**CEV (x10^3^)**		**IHD (x10^3^)**		**COPD (x10^3^)**		**LC (x10^3^)**		**LRI (x10^3^)**		**All (x10^3^)**	
Age	Pres.	Fut.	Pres.	Fut.	Pres.	Fut.	Pres.	Fut.	Pres.	Fut.	Pres.	Fut.
25–29	0 (0.0–0.2)	0 (0.0–0.2)	1 (0.6–1.2)	1 (0.5–1.1)	0 (0.0–0.1)	0 (0.0–0.1)	0 (0.0–0.1)	0 (0.0–0.1)	0 (0.0–0.8)	0 (0.0–0.5)	5 (3.6–5.3)	4 (3.2–4.8)
30–34	1 (0.4–1.1)	0 (0.0–0.5)	2 (1.5–2.3)	2 (1.2–1.1)	0 (0.0–0.1)	0 (0.0–0.1)	0 (0.0–0.2)	0 (0.0–0.2)	1 (0.9–1.3)	1 (0.8–1.1)	8 (6.6–9.5)	6 (5.2–7.7)
35–39	1 (0.7–1.2)	1 (0.6–1.1)	4 (3.0–4.3)	3 (2.6–3.6)	0 (0.0–0.2)	0 (0.0–0.2)	0 (0.0–0.2)	0 (0.0–0.2)	2 (1.3–2.1)	1 (1.0–1.5)	13 (10.6–15.5)	11 (9.0–13.2)
40–44	1 (0.9–1.6)	1 (1.0–1.4)	6 (5.3–7.3)	6 (4.6–6.3)	0 (0.0–0.2)	0 (0.0–0.2)	1 (0.5–1.1)	1 (0.4–1.0)	2 (1.4–2.1)	2 (1.3–2.0)	18 (14.8–21.4)	16 (13.2–19.1)
45–49	2 (1.9–2.7)	2 (1.5–2.1)	13 (10.1–13.8)	9 (7.6–10.4)	0 (0.0–0.3)	0 (0.0–0.2)	2 (1.3–2.1)	1 (0.9–1.4)	2 (1.8–2.4)	2 (1.3–2.0)	28 (23.0–33.4)	22 (18.0–26.4)
50–54	4 (3.1–4.2)	3 (2.4–3.4)	21 (16.8–23.0)	15 (13.4–18.4)	1 (0.5–1.1)	1 (0.4–1.0)	3 (2.7–3.7)	3 (2.5–3.3)	2 (2.0–3.2)	2 (1.7–2.3)	44 (36.0–52.4)	35 (27.8–40.6)
55–59	5 (4.5–6.1)	5 (4.3–5.9)	30 (24.3–33.3)	29 (23.5–32.2)	1 (0.9–1.3)	1 (0.9–1.3)	5 (4.7–6.4)	6 (4.9–6.6)	3 (2.5–3.5)	3 (2.4–3.2)	62 (49.9–73.0)	59 (47.6–69.3)
60–64	8 (6.4–8.7)	11 (9.3–11.9)	42 (34.4–47.2)	56 (46.2–63.3)	2 (1.8–2.4)	3 (2.4–3.2)	8 (6.6–9.0)	10 (8.4–11.5)	3 (2.6–3.6)	4 (3.4–4.7)	81 (65.5–95.5)	105 (84.5–123.1)
65–69	8 (6.9–9.4)	14 (11.9–16.0)	41 (33.6–46.0)	67 (55.4–75.9)	3 (2.1–3.2)	4 (3.2–4.4)	7 (6.5–8.9)	11 (9.2–12.7)	3 (2.2–3.1)	4 (3.4–4.6)	82 (66.2–96.7)	130 (104.2–151.8)
70–74	12 (11.1–14.6)	18 (15.3–20.6)	59 (48.7–66.7)	82 (67.2–92.0)	4 (3.4–4.6)	6 (4.5–6.3)	8 (7.0–9.5)	11 (9.2–12.8)	3 (2.9–4.2)	5 (3.9–5.4)	113 (91.8–133.7)	156 (126.7–184.1)
75–79	15 (12.9–17.2)	21 (18.5–25.3)	62 (50.4–69.0)	104 (85.7–117.3)	4 (3.6–4.9)	7 (6.0–8.2)	5 (4.7–6.4)	10 (7.9–10.8)	4 (3.1–4.3)	6 (5.1–7.0)	118 (95.9–140.0)	198 (160.0–235.1)
80plus	33 (27.6–37.7)	78 (64.9–89.3)	154 (126.8–173.7)	372 (319.8–425.5)	14 (11.1–16.0)	31 (27.2–36.2)	7 (5.7–7.8)	17 (14.3–19.6)	16 (14.2–19.6)	42 (34.4–47.2)	332 (270.0–392.5)	818 (662.2–964.7)
**TOTAL**	**90 (75.5**–**103.5)**	**154 (129.3**–**177.1)**	**435 (365.4**–**500.3)**	**746 (626.2**–**857.4)**	**29 (24.3**–**33.4)**	**53 (44.2**–**60.6)**	**46 (38.5**–**52.9)**	**70 (58.7**–**80.4)**	**41 (34.3**–**47.2)**	**72 (60.3**–**82.8)**	**904 (733.1**–**1,067.8)**	**1560 (1,260.0**–**1,840.0)**

Elderly people are more sensitive to air pollution, which will result in a large increase of the excess mortality rate in the future compared to the present scenario. Silva et al. (2016) estimated a decrease in the number of premature deaths for a future RCP8.5 scenario in Europe of around 150,000 deaths less that for the present period (2100 vs. 2000). However, this work used a similar y_0_ for the baseline mortality in the entire domain. Hence, the differences between this previous work and this contribution could be attributed to several causes, including the different use of baseline mortality (here three different baselines are used for Central, Western and Eastern Europe), a different reference year, the increased resolution used here -with air quality data from regional simulations- or to the fact that Silva et al. (2016) did not take into account the projected population, as done in this contribution.

Over Western Europe, Fig. 7 shows an increase of the mortality under the future scenario since pollution and population increase. Albeit a decrease of the pollution over Central Europe is expected, an important aging of the population is projected by the UN in this area, together with an increase of urban population, which will lead to an increase in the premature mortality associated to air pollution. The opposite behaviour is observed over the Eastern region, where the population will decrease and the pollutant concentration will increase, resulting in an increase of the mortality rate.

Table 3 indicates that all the excess mortality rates follow the same pattern (a significant increase with the age of the group in both the present and future scenarios). LRI keeps a similar number of deaths related to air pollution until the group 80+, when the mortality rate associated to PM_2.5_ increases considerably. Hence, LRI is strongly dependent on the increase of population in the range 80+, that will increase from 4% in the present period to 9% in the future projections. LRI increases from a contribution to the total excess mortality rate of 41,000 to 72,000 (95% CI 60,300–82,800) in the future RCP8.5 scenario.

Also, the excess LC mortality rate associated to PM_2.5_ is to be highlighted: 46,000 (95% CI 38,500–52,900) in the present vs. 70,000 (95% CI 58,700–80,400) in the future scenario. For this pathology the mortality rate increases with the age up to a maximum for the age range 70–74. From there, the mortality rates decrease again. This fact could be ascribed to the fact that the impacts of lung cancer on mortality are not immediate, as in other endpoints like IHD or CEV. Henceforth, people affected by lung cancer at such advanced age may die from another pathology instead of LC.

**Fig. 8:**
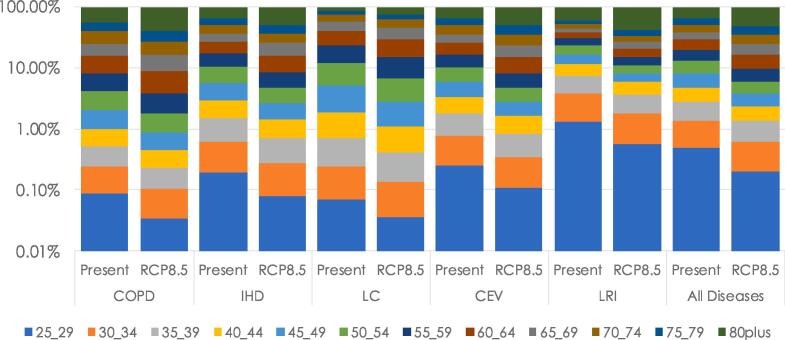
Present (1991–2010) mortality and future (RCP8.5, 2031–2050) estimation of deaths per pathology and age group, shown in %.

Finally, it is also noteworthy that the premature mortality from 25 until 60 years decreases in the future for all the endpoints. From 60 years old, mortality rates increase in comparison with the present period due to the aging of the population in the future. It should be born in mind that, according to the available literature, the RRs are constant for the different age groups considered in this contribution for COPD, LC and LRI, while epidemiologic studies of risk factors indicate that IHD and CEV RRs decline with the logarithm of age (Glymour et al., 2008, Krewski et al., 2009, Pope et al., 2009, Singh et al., 2013, Burnett et al., 2014). RRs for IHD and CEV also decrease when age increases. Similar patterns were obtained by Burnett et al., 2014, Cohen et al., 2017, Burnett et al., 2018. Henceforth, despite the RRs decrease with age, the total contribution to the excess mortality associated to air pollution in the future scenarios must be sought in the modifications in the population pyramid (Fig. 2). The 60–64 age group largely increases in the future and despite the reduction of the RRs, this group presents the largest mortality rate for the future period for people over 45 (Table 3 and Fig. 8).

## 4 Discussion and conclusions

The estimation of the contribution of fine particulate matter to the premature mortality rate over Europe leads to a total of 904,000 per year over Europe during the present period (1991–2010). Taking into account future potential changes in (1) population according to the UN projections and (2) climate penalty under the RCP8.5 scenario, this number is projected to increase (under the aforementioned hypothesis) by 73% for the future period 2031–2050 (1,560,000). This occurs despite the total population for Europe slightly decreases (808 million for present estimations vs. 806 million for 2050 s UN-based population). However, a large increase in the number of aged dwellers leads to nearly doubling the excess mortality rate associated to PM_2.5_ estimated for the year 2050.

The areas with the largest annual mortality rates are large European cities, Central and Eastern Europe. The main causes of mortality in Europe related to fine particles are cardiovascular diseases (Ischemic Heart, IHD, and Cerebrovascular Diseases, CEV), representing 58% of the excess premature mortality over the target area. These pathologies are distributed throughout the whole domain, particularly IHD. IHD and CEV are more sensitive to the levels of air pollution than the other endpoints. In addition, IHD is the pathology leading to the highest mortality rates associated to air pollution in Europe, accounting for an excess mortality rate of 435,000 per year during the present period and increasing to 746,000 under the future RCP8.5 scenario for 2031–2050 when considering the projected population, especially the aging of the European dwellers.

Albeit the population and PM_2.5_ decreases in certain areas of Europe from the present (1991–2010) to the future (2031–2050) periods, the total premature mortality rate will increase in the future because of two main reasons: (1) the increase in the most sensitive population group (elderly dwellers), according to the UN 2050 population projections; and (2) the increase in the population for the year 2050 over those regions where the PM2.5 concentration will also increase (central and southern Europe).

This contribution has focused on the impact from future changes in PM_2.5_ levels linked to climate changes together with projections of the European population. Understanding the sensitivity to these parameters is crucial in order to improve the assessments of future premature mortality associated to particulate matter. However, the future PM_2.5_ levels will to a large degree be driven by changes in the anthropogenic emissions both within and outside Europe. Previous studies (typically with a lower resolution in the models applied) have shown that the expected decrease in the European emissions will lead to an overall decrease in the levels of fine particles across Europe and that the impact from climate penalty is less relevant than the mitigation scenarios forecasted for Europe (Doherty et al., 2017; Cholakian et al., 2019). In this sense, in terms of health impacts, Geels et al. (2015) found a large decrease in the future number of premature deaths in Europe due to emissions changes under the RCP4.5 scenario. Therefore, further research should be devoted to the high-resolution assessment of air pollution mitigation scenarios and their impacts on human health.

The methodological approach used in this contribution (using detailed information about baseline mortality coming from regional European information, high-resolution PM_2.5_ data as well as projected future population) can help providing a more consistent approach to the estimation of air pollution-derived health issues in each European region, since the different baseline mortality takes into account different socio-economical and climatic conditions from each region. The findings presented here underline the importance of a continuous focus on regulation of air pollution as both climate change and the ageing of population will potentially counteract benefits of air pollution policies.

**Authorship contributions**

Conception and design of study, acquisition of data: Patricia Tarín-Carrasco, Ulas Im and Pedro Jiménez-Guerrero. Analysis and/or interpretation of data: Patricia Tarín-Carrasco, Laura Palacios-Peña and Pedro Jiménez-Guerrero. All authors should have made substantial contributions to all of the following: (1) drafting the article or revising it critically for important intellectual content, (2) final approval of the version to be submitted.

**Data availability**

All the data included in this contribution is available by contacting the corresponding author (pedro.jimenezguerrero@um.es).

## Declaration of Competing Interest

The authors declare that they have no known competing financial interests or personal relationships that could have appeared to influence the work reported in this paper.
