# Supplementary material for: Contribution of fine particulate matter to present and future premature mortality over Europe: A non-linear response
Source: Environ Int. 2021 Aug;153:106517. doi: 10.1016/j.envint.2021.106517 (PMC8140409; doi:10.1016/j.envint.2021.106517)
Supplement: Supplementary Data 1 [file mmc1.docx]

**Supplementary Material to:**

**Contribution of fine particulate matter to present and future premature mortality over Europe: a non-linear response**

Patricia Tarín-Carrasco^1^, Ulas Im^2^, Camilla Geels^2^, Laura Palacios-Peña^1,⊗^ and Pedro Jiménez-Guerrero^1,3,*^

^1^ Department of Physics, Regional Campus of International Excellence Campus Mare Nostrum, University of Murcia, 30100 Murcia, Spain

^2^Aarhus University, Department of Environmental Science, Frederiksborgvej 399, DK-4000, Roskilde, Denmark.

^3^Biomedical Research Institute of Murcia (IMIB-Arrixaca), 30120 Murcia, Spain

^⊗^Now at: Dept. of Meteorology, Meteored, 30893 Almendricos, Spain

*Corresponding author: pedro.jimenezguerrero@um.es

**SM.1 Integrated Exposure-Response (IER) model and Global Exposure Mortality Model (GEMM) methodologies.**

The excess premature mortality rates in Europe have been estimated by using exposure-response functions. Different methodologies, scenarios, periods and/or emissions are used in the scientific literature to estimate the impacts of outdoor pollution on human health.

Equation SM.1 describes the exposure-response function followed in this work. This equation is based on epidemiological relationships between air pollution concentration and mortality and it is applied in each grid cell where information about air pollution and population is available (Lelieveld et al., 2015, 2019):

ΔM = y_0_ [(RR-1) / RR] Pop (Eq. SM.1)

where *ΔM* is premature mortality for each disease; RR is the risk ratio, which estimates the relative risk of exposure to a specific pollutant (see Equations SM.2 and SM.3 for further details); *y_o_* is the baseline mortality rate for the year 2010; and *Pop* refers to the exposed population. *y_o_* varies for each mortality endpoint, age and European region and is estimated annually by the World Health Organization for each gender (GHDE, 2019).

In the present work, two different non-linear functions were a priori considered for the calculation of premature mortality due to exposure to PM_2.5_: the Integrated Exposure-Response (IER) model used by the Global Burden Disease (GBD); and the Global Exposure Mortality Model (GEMM) methodologies.

The IER methodology determines the risk ratios (RRs) values for each pathology and age range by using the following expression (Equation SM.2):

*RR = 1 + α {1 – exp [-ɣ (z-z_o_)^δ^] }* (Eq. SM.2)

A Monte Carlo method has been applied to determine *α, ɣ* and *δ* for each endpoint and age range (Burnet et al., 2018). *z* refers to the PM_2.5_ concentration in µg m^-3^ and *z_o_* is the concentration threshold for PM_2.5_, below which no risk is assumed for human health. *α, ɣ*, *δ* and *z_o_* were obtained from the Global Burden Disease study (GBD, 2017).

In the second methodology, coming from the Global Exposure Mortality Model (GEMM) (Burnett et al., 2018), RRs are calculated through a number of hazard ratio functions. These functions are based on 41 cohort studies from 16 countries and model the association between PM_2.5_ and non-accidental mortality. Hence, the methodology of Burnett et al. (2018) as implemented in Lelieveld et al. (2019) gives the following expression (Equation SM.3):

*RR = exp{θlog[(z/α)+1]/(1+exp{-(z-µ)/ν})}, where z=max (0, PM_2.5_-2.4 μg m^-3^)* (Eq. SM.3)

where *θ, α, µ* and *ν* are variables obtained from Burnett et al. (2018) and *z* is the PM_2.5_ concentration. Concentrations below a certain threshold (2.4 μg m^-3^) are not taken into account because of the high uncertainty associated to the limited PM data available from cohort studies below this value.

With respect to the studied endpoints, Lung Cancer (LC), Chronic Obstructive Pulmonary Disease (COPD), Cerebrovascular Disease (CEV), Ischemic Heart Disease (IHD) and Lower Respiratory Infection (LRI) were studied using IER methodology. The category of non-accidental diseases, which was defined as NCD+LRI; and the so-called ‘other NCDs’, defined as the subtraction of the above categories to NCD+LRI, were added when using GEMM.

**SM.2 Comparison of IER vs. GEMM methodologies**

The following SM section shows the comparison between the two methodologies described above, IER and GEMM. Both methodologies use the same baseline data, population and concentration of PM_2.5_ for the estimation of the premature deaths associated to each pathology described in the Sections 2.2 and 2.3 of the manuscript.

These concentrations are used as input to Equations SM.2 and SM.3 in order to estimate the RRs, and then to Equation SM.1 for the estimation of the excess premature mortality. The differences between SM.2 and SM.3 equations are caused by the different number of cohort studies included in each methodology. The number of excess premature deaths associated to air pollution resulting from Equation SM.1 in the present period (1991-2010) estimated with IER and GEMM methodologies are shown in Table SM.1 and Figure SM.1.

Table SM.1. Excess premature mortality Europe due to air pollution and percentage of each cause estimated by IER and GEMM for present period (1991-2010). Pathologies have been selected so they are comparable between the two methodologies. Numbers in parenthesis represent the 95% confidence interval.

|  | **IER** | | **GEMM** | |
| --- | --- | --- | --- | --- |
|  | Cases (x10^3^) | % | Cases (x10^3^) | % |
| LRI | 7 (5.9-8.1) | 1.5 | 42 (35.3-48.3) | 6.7 |
| LC | 18 (15.1-20.7) | 3.8 | 48 (40.3-55.2) | 7.6 |
| COPD | 10 (8.4-11.5) | 2.0 | 28 (23.5-32.2) | 4.4 |
| IHD | 344 (289.0-396.0) | 72.1 | 424 (356.2-487.6) | 67.5 |
| STROKE | 98 (82.3-112.7) | 20.6 | 87 (73.0-100.0) | 13.8 |
| **TOTAL** | **477 (400.6-549.0)** | **100** | **629 (528.4-723.4)** | **100** |

Figure SM.1. Estimation of the causes of excess mortality per year (present period, 1991-2010) for IER (blue) and GEMM (yellow) for premature deaths associated with LRI, COPD, LC, STROKE and IHD over Europe.

The most noticeable difference is that GEMM estimates 150,000 more premature deaths per year due to PM_2.5_ in Europe than IER (629,000; 95% CI 528,400-723,400 vs. 477,000; 95% CI 400,600-549,000. The main differences are found over Eastern and Western European regions (agreeing with the most polluted regions by particles in the present), with minor differences found for Central Europe (Figure SM.2). The largest differences are seen for some hotpots coinciding with the largest European cities. The area of southern United Kingdom also stands as the area with the highest differences, with nearly 0.05 cases per km^2^ for GEMM.


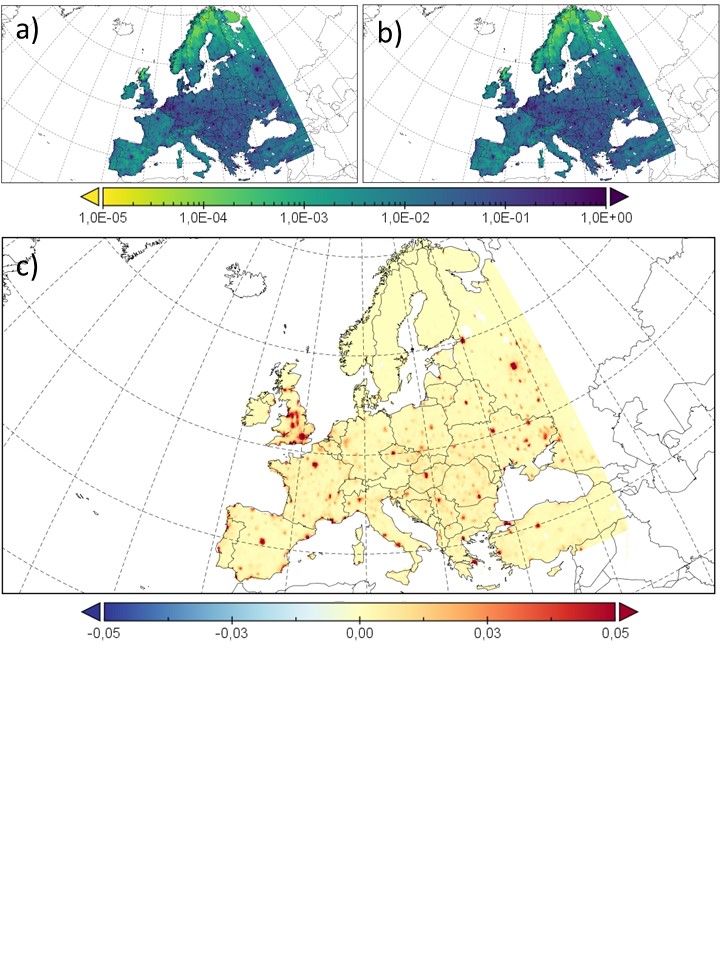


Figure SM.2. Annual premature deaths estimated with the (a) IER and (b) GEMM methods. (c) Difference in the number of premature deaths per year (all units: premature deaths per km^2^) between GEMM and IER methodologies.

Although the percentages of deaths associated with each mortality are similar for IER and GEMM (Table SM.1), the former methodology estimates more excess premature deaths per year related to COPD than to LRI (10,000; 95% CI 8,400-11,500 vs. 7,000; 95% CI 5,900-8,100, respectively, in IER) while GEMM shows an opposite behaviour (28,000 premature deaths associated to fine particles for COPD; 95% CI 23,500-32,200 vs. 42,000 for LRI, 95% CI 35,300-48,300). The IER results presented in this contribution are slightly higher than those obtained by Lelieveld et al. (2015), who estimate a total of 375,000 deaths for a similar domain over Europe and during a similar period.

Total number of excess premature deaths is 477,000 (95% CI 400,600-549,000) per year in the present study, that is, ca. +20% higher than the aforementioned work. Comparing the results for each pathology, Lelieveld et al. (2015) estimate 1,000 deaths for LRI (7,000 in this contribution, 95% CI 5,900-8,100); LC for 27,000 (18,000 here, 95% CI 15,100-20,700); COPD for 13,000 (10,000 in this contribution, 95% CI 8,400-11,500); IHD for 239,000 (344,000 here, 95% CI 289,000-396,000) and CEV for 95,000 (98,000 in this contribution, 95% CI 82,300-112,700). That is, the main difference in the total mortality rates associated to fine particulate matter comes from the estimation of premature deaths associated to ischemic heart disease (+30% in this contribution).

**SM.3 Time evolution of premature deaths in the 1991-2010 period estimated with the GEMM methodology**

In order to have an estimation of the impact of population growth, baseline mortality (Tabla SM.2) and population age on the change in premature mortality/year from 1991-2010, Figure SM.3 (left) shows a trend for increase of the European population included in the target domain for the 1991-2010 period, varying from 792 500 pop. in the year 1991 to 807 500 pop. in the year 2010. This involves an increase of 2% in the total European population. When focusing on total premature deaths (Figure SM.3, right), there is not a clear trend in the evolution of excess premature deaths per year associated to PM2.5 in the 1991-2010. The numbers at the right of the bars represent the variation of premature deaths each year with respect to the average of the target time period. The Figure SM.3, right indicates that the excess premature deaths vary from 862,900 (95% CI 698,700-1,018,000) premature deaths per year in the year 1991 to 882,400 (95% CI 715,500-1,042,000) premature deaths per year in 2010, ranging from 825,000 (95% CI 668,000-1,041,000) to 921 500 (95% CI 746,000-1,088,000) in the year 2004. Therefore, the estimation of 904,000 (95% CI 733,100-1,067,800) presented in the results of the manuscript, estimated from averaging the exposure over the 20-years period can be considered as representative of the target period, since no large variations are obtained in the years 1991-2010.

Table SM.2. Baseline mortalities (y_0_) used for estimating the premature deaths in the period 1991-2010 (GHDE, 2019).

| **Year** | West EU | Central EU | Eastern EU |
| --- | --- | --- | --- |
| 1991 | 928.18 | 955.19 | 994.36 |
| 1992 | 919.34 | 967.46 | 1051.74 |
| 1993 | 923.40 | 966.02 | 1192.25 |
| 1994 | 915.13 | 971.62 | 1282.00 |
| 1995 | 919.29 | 989.67 | 1268.87 |
| 1996 | 917.08 | 993.30 | 1218.35 |
| 1997 | 907.99 | 1001.61 | 1170.86 |
| 1998 | 908.46 | 984.41 | 1152.81 |
| 1999 | 906.88 | 985.73 | 1228.26 |
| 2000 | 896.04 | 976.25 | 1273.39 |
| 2001 | 888.55 | 979.02 | 1292.83 |
| 2002 | 890.77 | 990.26 | 1331.42 |
| 2003 | 891.69 | 1000.65 | 1351.17 |
| 2004 | 863.81 | 998.31 | 1346.12 |
| 2005 | 858.82 | 1015.42 | 1395.70 |
| 2006 | 852.55 | 1017.63 | 1324.80 |
| 2007 | 854.15 | 1024.55 | 1300.60 |
| 2008 | 859.06 | 1030.54 | 1310.89 |
| 2009 | 860.72 | 1040.88 | 1260.57 |
| 2010 | 858.82 | 1041.42 | 1266.08 |

Figure SM.3. (Left) Evolution in the European population in the target domain (in thousands). (Right) Annual premature deaths estimated with GEMM method for the period 1991-2010. This estimation includes the impact of population, baseline mortality and year-to-year varying air pollution in the 1991-2010 period.

**References**

Burnett, R., Chena, H., Szyszkowicza, M., Fann, N., Hubbell, B., Pope III, C. A., Apte, J. S., Brauer, M., Cohen, A., Weichenthal, S., Coggins, J., Di Q., Brunekreef B., Frostad, J., Lim, S. S., Kan, H., Walker, K. D., Thurston, G. D., Hayes, R. B., Lim, C. C., Turner, M. C., Jerrett, M., Krewski, D., Gapstur, S. M., Diver, W. R., Ostro, B., Goldberg, D., Crouse, D. L., Martin, R. V., Peters, P., Pinault, L., Tjepkema, M., van Donkelaar, M., Villeneuve, P. J., Miller, A. B., Yin, P., Zhou, M., Wang, L., Janssen, N. A. H., Marra, M., Atkinson, R. W., Tsang, H., Thach, T. Q., Cannon, J. B., Allen, R. T., Hart, J. E., Laden, F., Cesaroni, G., Forastiere, F., Weinmayr, G., Jaensch, A., Nagel, G., Concin, H. Spadar, J. V., 2018. Global estimates of mortality associated with longterm exposure to outdoor fine particulate matter. PNAS, 38 (115), 9592–9597. https://doi.org/10.1073/pnas.1803222115.

GBD, 2017. Causes of Death Collaborators. Global, regional, and national age-sex specific mortality for 264 causes of death, 1980-2016: a systematic analysis for the Global Burden of Disease Study 2016. Lancet, 390(10100), 1151–1210. https://doi.org/10.1016/S0140-6736(17)32152-9.

GHDE, 2019. Global Health Data Exchange. http://ghdx.healthdata.org/, last access: 10-12-2019

Lelieveld, J., Evans, J. S., Fnais, M., Giannadaki, D., Pozzer, A., 2015. The contribution of outdoor air pollution sources to premature mortality on a global scale. Nature, 525, 367–371. https://doi.org/10.1038/nature15371.

Lelieveld, J., Klingmüller, K., Pozzer, A., Pöschl, U., Fnais, M., Daiber, A., Münzel, T., 2019. Cardiovascular disease burden from ambient air pollution in Europe reassessed using novel hazard ratio functions. Eur. Heart J., 40, 1590-1596. https://doi.org/10.1093/eurheartj/ehz135.
